# Physical and Psychological Factors Contributing to Incidental Falls in Older Adults Who Perceive Themselves as Unhealthy: A Cross-Sectional Study

**DOI:** 10.3390/ijerph18073738

**Published:** 2021-04-02

**Authors:** Mikyong Byun, Jiyeon Kim, Ji Eun Kim

**Affiliations:** 1 BK21FOUR R&E Center for Learning Health Systems, College of Nursing, Korea University, Anam-dong, Seongbuk-Gu, Seoul 02841, Korea; mulanbb@korea.ac.kr (M.B.); tortoi@korea.ac.kr (J.K.); 2 Department of Nursing Science, College of Medicine, Catholic Kwandong University, 24 Beomil-ro, 579beon-gil, Gangneung-si, Gangwon-do 25601, Korea; 3 Department of Neurology, Gangneung Asan Hospital, University of Ulsan College of Medicine, 38 Bangdong-gil, Sacheon-myeon, Gangneung-si, Gangwon-do 25440, Korea

**Keywords:** public health, fall, self-reported health status

## Abstract

Falls have become one of the common causes of morbidity and mortality in the elderly. Advanced age is a strong predictor of falls. Additionally, those who perceive themselves as unhealthy are more likely to suffer from incidental falls in accordance with aging. We aimed to compare individual, physical, and psychological factors between older adults with and without a fall history. Then, we tried to identify physical and psychological variables associated with falls by controlling for individual characteristics. We analyzed public data from the 2017 National Survey of Older Persons in South Korea. Seniors aged 65 years and over who considered themselves in poor health status were eligible. A total of 2765 women and 1280 men (*n* = 4045) were enrolled, and 940 adults suffered a fall within a year (the average number of falls was 2.5). We applied individual variable-adjusted models and discovered that hearing discomfort (odds ratio (OR) 1.30, 95% confidence interval (CI) 1.09–1.55), limited activities of daily living (ADL) (OR 1.40, 95% CI 1.13–1.74), limited instrumental activities of daily living (IADL) (OR 1.34, 95% CI 1.13–1.61), and depression (OR 1.44, 95% CI 1.23–1.69) significantly increased risk for falls on multivariate logistic regression. Our findings suggest that hearing discomfort, limited ADL/IADL, and depression need to be addressed observantly to prevent falls in the elderly who consider themselves unhealthy.

## 1. Introduction

We are on the verge of “homo-hundred era” [1], which is highly interlinked to an increased number of senior populations aged 65 and over. The rapidly growing number of the aged population also matters because it provokes many socioeconomic, individual, and emotional conflicts [2]. South Korea is one of the world’s fastest aging countries. According to an Organisation for Economic Co-operation and Development (OECD) report, our society has become an aged society just 17 years after it officially reached the aging society in 2000 [3]. In 2019, older adults aged 65 and over accounted for more than 15% of the total population. An official study by Statistics Korea predicted that our nation will become the world’s most aged society by 2067, with the elderly population composing about 46% [4]. Life expectancy went up by more than 30 years over the past century. This means that more people are at risk of living with multiple and complicated chronic illnesses as time goes by. Compared with other age groups, older adults have unique characteristics. They are quite vulnerable to physical or psychological stressors and suffer from gradual losses of multi-organ functions. These features give rise to various degenerative diseases, cardiopulmonary diseases, cancers, strokes, and even falls.

Older people, even without any specific diseases, are prone to experience accidental falls, as physical or psychological impairments originate from the inevitable aging process [5]. Falls cause morbidity and mortality in the elderly. It also results in enormous financial and health care burdens on society and the individual [5,6]. The estimated annual fall-related expense for 60-year-old or older Koreans was estimated at South Korean Won (KRW) 1.38 trillion, which is equivalent to USD 1 billion [7].

Understanding self-rated health (SRH) as an indicator for forthcoming morbidity and mortality, particularly in the older population, is clinically important [8,9,10,11,12]. In general, SRH is a practical and relatively reliable tool for measuring respondent’s general health status [13,14]. It integrates physical and psychological features, along with daily life satisfaction, overall sense of well-being, and quality of life [15,16,17]. The “negative” self-rated health status in older people does not simply reflect their physical discomfort or pain. It encompasses demographic, psychological, affective, and social conditions of the individual. Previous studies reported that negative SRH was in close relationship with a higher prevalence of chronic diseases, mortality, and hospital visits [18]. Other longitudinal studies also revealed that negative SRH correlated with unfavorable health outcomes such as functional limitations and consequent falls [19].

Not all the older people who regard themselves as unhealthy experienced falling incidents. However, it is very important to find relevant factors between fallers and non-fallers with the same negative SRH. This study aimed to identify individual, physical, and psychological factors affecting falls in negative SRH adults. Further, we tried to identify statistically significant physical and psychological variables associated with falls by controlling for individual characteristics.

## 2. Materials and Methods

### 2.1. Source of Data

Under the approval of the National Statistical Office, we obtained official data from the 2017 National Survey of Older Persons (NSOP) [20]. The database consists of stratified random samples of 10,000 people who were dwelling in housing facilities and was devised to represent the Korean elderly population. This NSOP, using in-person interview, was conducted on 10,299 senior citizens (aged 65 years or more, including 226 representatives) from June 2017 to August 2017 in 934 survey areas. Statistics Korea approved an alteration of questionnaire design following expert reviews and pretests (Authorization No.11771). A total of 60 specialized surveyors, trained by the skilled research staff in advance, conducted the survey (15 teams each consisting of four surveyors and one supervisor).

### 2.2. Study Design and Subjects Selection Criteria

**Figure 1:**
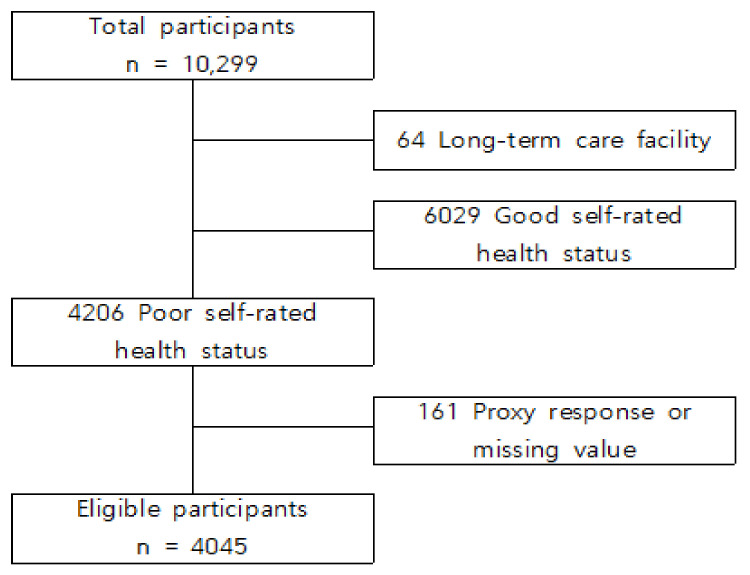
Consort flow diagram of study population inclusion.

Adults aged 65 or more who met the following inclusion criteria were enrolled: (a) poor self-rated health status; (b) community dwelling; and (c) without proxy response or missing data. Consequently, 4045 participants with negative SRH were eligible (Figure 1). Self-rated health status was estimated by using the five-choice Likert scale question, “How would you judge your health status generally?” The five choices were dichotomized into “negative” (very poor or poor) or “positive” (fair, good, or very good) for analysis.

Based on cross-sectional, descriptive, and correlational study design, we assessed predictive factors for falls using three (individual, physical, and psychological) pivotal categories. Next, we built individual variables-adjusted models to figure out crucial physical and psychological factors that contribute to incidental falls on multiple logistic regression.

### 2.3. Key Measurements

#### 2.3.1. Falls

Fall history was preferentially evaluated using a single question: “Have you experienced a fall (stumble, slip, or fall) within a year?” The participants could respond whether they “experienced a fall” or “did not experience a fall.” Those who responded “yes” to the above were designated as fallers, in which case an additional answer to the number of falls within 12 months was obtained.

#### 2.3.2. Individual Factors

Individual variables were grouped into four subcategories—demographic, socioeconomic, health status, and health-related behavior. Firstly, demographic factors were composed of age, sex, marital status (living with or without spouse), and living status (alone, living with spouse, or living with children or others). Secondly, education level (0–6 years, 7–9 years, 10–12 years, or ≥13 years) and degree of household income (Q1 [the lowest quartile], Q2, Q3, Q4, or Q5) of respondents were classified as socioeconomic variables. Thirdly, health status variables included chronic medical diseases (hypertension, diabetes, dementia, and arthritis), calculated body mass index (BMI), and the number of current medications (0, 1, 2, or ≥3). Lastly, health-related behaviors involved exercise, smoking (past/never or current), and drinking.

The presence or absence of chronic illness was determined using the following questions, “have you been suffering from any diseases such as hypertension, diabetes mellitus, arthritis or dementia for more than three months?”and “have you been diagnosed with the above-mentioned diseases by any doctor?” Subjects who replied “yes” to both were categorized to have a chronic illness. The number of current medications was identified using the next question, “how many prescribed medications have you been taking over the past three months or more?” Exercise for more than 150 min per week is recommended by the World Health Organization (WHO) [21]. Response of “no” to “do you usually exercise?” question was sorted as “none.” Therefore, exercise levels were classified into three subtypes—within the recommended level (≥150 min/week), below the recommended level (<150 min/week), and none. Alcohol intake was evaluated using the National Institute on Alcohol Abuse and Alcoholism criteria [22]. In older adults aged 65 years and over, alcohol intake of less than one standard drink (a 350 mL glass of beer) per day was regarded as an adequate amount in this study, and consuming more than one standard drink per day was considered excessive. “None” was assigned to those who did not drink at all.

#### 2.3.3. Physical Factors

Physical variables involved hearing difficulty, visual difficulty, limitation of activities of daily living (ADL), and limitation of instrumental activities of daily living (IADL).

Sensory discomforts were divided into visual and auditory discomforts. Visual discomfort was defined as “uncomfortable” in individuals who responded “uncomfortable” or “very uncomfortable.” Additionally, hearing discomfort was defined as “discomfort” in those who answered “uncomfortable” or “very uncomfortable.”

The ADL limitation was assessed according to the Korean Activities Daily Living Scale [23]. This scale consists of seven domains, i.e., “bowel and bladder control (continence)”, “toilet use”, “getting up and walking across the room (transfer)”, “eating food”, “bathing”, “face washing, brushing teeth, and shampooing”, and “dressing”. Each domain is estimated by a three-point rating method (total independence/partial dependence/total dependence) and a higher score implies more severe limitations. Score 0, i.e., “total independence,” was determined as “no limitation.” Score 1 and 2, i.e., “partial and complete dependence,” were determined as “having limitation.” Respondents who reported any dependency in more than one domain were regarded as having ADL impairment.

The presence of IADL limitation was estimated based on the Korean Instrumental Activity of Daily Living Scale [23]. This scale consists of 10 items, namely, “grooming”, “housework”, “preparing foods”, “doing laundry”, “taking medicines on time”, “going out for a short trip”, “shopping”, “managing money”, “ability to use the telephone”, and “using public transportation.” Total independence was regarded as “no limitation.” By contrast, the others (partial, complete, little, much dependence, and cannot be conducted at all) were classified as “having limitation.” Additionally, respondents who claimed any restriction in more than one item were regarded to have an IADL limitation.

Nutritional status was estimated in accordance with the Determine Your Nutritional Health questionnaire of the Nutrition Screening Initiative [24]. This tool is composed of 10 binary (yes or no) responses. To each question, a “yes” is scored in a range of one to four, whereas “no” is scored zero. The total scores of 10 items are classified as follows: 0–2, good nutritional state; 3–5, having moderate nutritional risk; and ≥6, having high nutritional risk. That means a score of 0–2 was considered to be “in good nutrition,” while a score more than 2 was classified as “in poor nutrition.”

#### 2.3.4. Psychological Factor

In 2017 NSOP questionnaires, depression was the only interviewed psychological characteristic. Depression was assessed using the Korean version of the 15-item Geriatric Depression Scale (Short-version of GDS-K; SGDS-K) [25]. According to a previous Korean study, the optimal cut-off values for SGDS-K in screening for clinically meaningful depressive mood were proposed to be ≥8 (total scores range from 0 to 15). Based on this suggestion, scores of ≥8 and <8 were dichotomized into “depressed” and “not depressed” in our study.

### 2.4. Statistical Analysis

**Figure 2:**
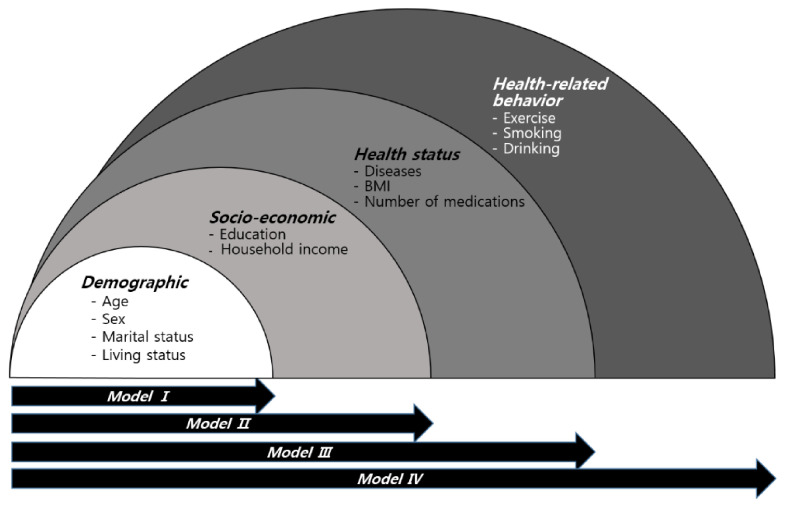
Proposed individual variables-adjusted models. The variables from individual subcategories (demographic, socioeconomic, health status, and health-related behavior) were merged in consecutive order and four experimental models were established.

We performed descriptive statistics using the χ^2^ or *t*-test to compare differences in fall-related experiences by individual, physical, and psychological variables (*p*-value less than 0.05 is statistically significant). Each independent factor was included for univariate logistic regression, from which statistically significant factors were selected for multivariate logistic regression. We then designed our unique individual variables-adjusted models in order to minimize potential confounding effects by individual factors. The variables from the individual category, including demographic, socioeconomic, health status, and health-related behavior subcategories, were combined in groups and entered into logistic regression models. In other words, we assumed that factors in demographic subcategories (such as age, sex, and marital status) were the least modifiable among the four subcategories. The other subcategories such as socioeconomic, health status, and health-related behaviors could be more easily modifiable (depending on one’s effort) in consecutive order. Base on this hypothesis, we invented Model I (consisting of demographic subcategory only) and we gradually encompassed easily modifiable subcategories in a stepwise way, which is presented as Model II, III, and IV. (Model I, demographic variables only; Model II, demographic and socioeconomic variables; Model III, demographic, socioeconomic, and health status variables; Model IV, all the individual variables) (Figure 2). Odds ratios (ORs) and corresponding 95% confidence intervals (CIs) were also presented. The statistical significance refers to a *p*-value less than 0.05. Data were analyzed using the IBM SPSS version 22.0 software package (IBM, Armonk, NY, USA).

## 3. Results

### 3.1. Fall Incidence and Average Number of Falls

Sample size calculation was based on the fall prevalence among community-dwelling older Koreans, by using a prevalence of 20.6% [26]. The margin of error was considered to be 5% and the non-responder rate to be 10%. The final estimated sample size was 263. In our study, among 4045 adults with negative SRH, 940 adults (23.2%) experienced a fall within a year. The average number of falls was 2.5 events per year.

### 3.2. Differences in Individual Characteristics between Groups with and without Fall History

Differences in fall history according to individual variables are summarized in Table 1. With respect to demographic, socioeconomic, health status, and health-related behavior components, more than half of the items (except education, diabetes, dementia, BMI, exercise, smoking, and drinking) were statistically significant between fall and non-fall groups.

### 3.3. Differences in Physical and Psychological Characteristics between Groups with and without Fall History

Statistically significant differences were observed between fall and non-fall groups in terms of physical characteristics such as visual, hearing, ADL, IADL, and nutrition status (Table 2). Visual or hearing discomforts, ADL or IADL restriction, and poor nutrition were more frequently observed in the fall group.

There was also a significant difference between the two groups in view of depression as a representative psychological factor (Table 2). More than half of non-fallers (62.6%) did not report depressed mood.

### 3.4. Multivariable Logistic Regression Analysis of Relevant Factors in Elderly People with Negative SRH Experiencing Falls Using Individual Variables-Adjusted Models

To evaluate the physical and psychological factors affecting risks for falls, we applied unique four individual variable-adjusted models, as stated above. In Model IV, our study showed that falls in old people who felt themselves unhealthy were significantly associated with hearing discomfort (odds ratio (OR) 1.30, 95% confidence interval (CI) 1.09–1.55), limited ADL (OR 1.40, 95% CI 1.13–1.74), limited IADL (OR 1.34, 95% CI 1.13–1.61), and depression (OR 1.44, 95% CI 1.23–1.69) on multivariate analysis (Table 3). Interestingly, poor nutritional status was found to be associated with falls in Model I and II only.

## 4. Discussion

Understanding the health status of older people is very important in terms of disease prevention, health promotion, and health care delivery services [27,28]. Assessing the health status of the elderly on a regular basis is often necessary to improve one’s health-related behavior and to prevent adverse events such as falls. In the field of health care for the elderly who already regard themselves as unhealthy, it is essential to encourage them to lead ordinary lives as much as possible. Additionally, early detection and timely intervention to supplement their physical and psychological handicaps cannot be overemphasized.

Self-rated health status is considered a feasible and reliable indicator in estimating general health status because of its simple format of a five-point scale [13,14]. It incorporates diverse personal, social, and functional aspects of the individual. These aspects interact with the overall sense of well-being, concern for self-care, and compliance to the medical treatment [15,16,17]. Many studies suggested that SRH in older adults can serve as a powerful and sensitive predictor of adverse long-term health outcomes such as morbidity, disability, and premature mortality [8,9,10,11,12]. Thus far, underlying mechanisms that connect negative SRH to adverse health outcomes are not clear yet [29]. To elucidate this matter, several cross-sectional and longitudinal studies indicated low-grade systemic inflammatory processes as a possible cause [30,31,32,33]. Chronic and persistent elevation in serum pro-inflammatory cytokines eventually play a role in the development of age-related functional decrepitude, cardiovascular diseases, arthritis, diabetes, periodontitis, and even cancers [34]. As is well known, exposure to chronic inflammation is often accompanied by symptoms such as lethargy, loss of appetite, and passiveness [35]. These symptoms may adversely affect subjective health perceptions in those who do not suffer from any medical diseases [36].

Many researchers presented positive correlations between poor self-perceived health and increased fall risk. Shimada et al. validated fall risks with a subjective risk rating of specific tasks (SRRST) score, which evaluates a person’s ability to walk, toilet, and going up and down the stairs by a day-center staff. A higher score reflected poor individual health and it correlated with a history of falls in frail older adults [37]. Singh et al. analyzed the fall risk factors in 3935 adults (aged 55 and over) by measuring physical performance and self-rated health. Falls are correlated with diabetes, arthritis, urinary incontinence, poor self-rated health, and lower handgrip strength. Additionally, those who felt their health conditions were equal to or less than fair had a higher chance of falls [38]. In this study, we compared individual, physical, and psychological variables in “feeling unhealthy” for older adults with and without a fall history. As mentioned above, all the adverse physical and psychological factors were more frequently found in fallers with negative SRH. Meanwhile, about half of individual factors (such as education, diabetes, dementia, BMI, exercise, smoking, and drinking) did not show statistical significance between fall and non-fall groups in our study. Then, we tried to identify statistically significant physical and psychological risk factors contributing to incidental falls using stepwise individual variables-adjusted models. Our results demonstrated that hearing discomfort, limited ADL, limited IADL, and depression increased fall risks in older adults who subjectively perceive themselves as unhealthy.

Maintaining posture and balance require adequate interactions between diverse sensory inputs and integrated reactions to the continuous changes of surroundings [39,40]. Sensory impairments restrict one’s ability to monitor outer signals that help us adjust spatial orientations. Difficulty in hearing has been reported as a risk factor for falls [41,42]. Its prevalence increased with age despite controlling for noise exposure history [43]. As we age, our body gradually loses its unique ability to adapt to the altered sensory changes, and this is why we are prone to falls [44,45]. Additionally, there is an interesting report that audiology patients with hearing aids fell less often than those without [46]. In our study, hearing difficulty significantly increased fall risk on logistic regression models, whereas visual difficulty did not. This discrepancy might be attributable to differences between hearing and vision aid uses in our real life. Vision aids such as eyeglasses, magnifying glasses, and contacts are readily accessible and cheaper than hearing aids. For example, well-fitted visual aids provide good calibrations immediately after wearing them, but hearing aids take us time to be familiar with them. To make it worse, hearing aids users often complain about noises (such as buzzing or humming), which make it difficult to wear them regularly. In summary, seniors with sensory difficulties have a fair chance to experience accidental falls.

According to our survey in detail, most of the fallers pointed out environmental problems such as “slippery flow (26.4%)” or “high doorsill (16.5%)” as a cause (data not shown). We postulated that these findings may mirror the possible effects of the sensory impairments on functional balance in old adults. Hearing difficulty may also be linked to negative SRH because of hearing-related fatigue, mood change, and diminished social interactions [47,48,49,50]. Thus, providing timely support and regular feedback to compensate for the sensory difficulties is crucial in reducing fall risk and amending the self-feeling of unhealthiness.

Both ADL and IADL limitations were identified as significant fall risk factors in our analysis. Çinarli et al. reported that older adults at higher risk for falls were more dependent on daily living activities and complained of lower quality of life [51]. Measuring mobility pattern changes or locomotion variabilities in elderly people can be helpful in the elderly. Several studies on wearable sensors showed that these were feasible to monitor activities of community-dwelling older adults. Sensors provided sufficient information to assess mobility patterns and to estimate future falls in the elderly [52]. Of course, we should consider the fact that there is a possible bi-directional interplay between ADL/IADL limitation and falls. Limited ADL/IADL can also be a consequence of falls rather than a unilateral risk factor for falls.

In our study, depression was found to be another powerful risk factor in older adults with negative SRH. Depression and falls had a significant bi-directional complex interplay in a previous report [53]. In depressive older adults, somatic symptoms (such as poor appetite), weight loss, and nutritional deficiencies can lead to incidental falls. Depression is related to secondary cognitive deficits which are called depression-induced cognitive impairment. It affects mainly attention, frontal/executive function, and psychomotor speed [54]. Walking is a complex process requiring interactions among the motor system, sensory system, and cognitive brain function. Depression often interferes with our ability to react to environmental changes and to coordinate motor outputs [53,55]. On the other hand, antidepressants can also increase the risk for falls due to their side effects such as hypersomnolence or dizziness [56,57]. Health care providers should pay particular attention to depressive older adults who are taking antidepressants.

There were three limitations in our study. First, our analysis had a cross-sectional design, and this made it difficult to find the causality. In other words, the cause-and-effect relationship between falls and negative SRH might be ambiguous, which was an inherent limitation of the study design. Second, there may exist recall bias since the primary data source was interview responses. Third, depression was the only psychological factor included in the 2017 NSOP interview items. Our findings necessitate further validations by including fear of falling, anxiety, and other psychological factors.

Despite these limitations, our fall study was the first nationwide data of community-dwelling older adults who already regard themselves as unhealthy. Additionally, we also investigated physical and psychological factors associated with incidental falls using multi-step variable-adjusted models. Based on our findings, public health professionals may gain insight on how important it is to detect fall-prone older adults early and provide timely support to relieve individual, social, and worldwide burdens from incidental falls.

## 5. Conclusions

Falls are an important public health issue among the elderly population worldwide. Our findings suggested that physical and psychological factors, especially auditory discomfort, limited ADL/IADL, and depression, need to be addressed to prevent falls in older adults who regard themselves as unhealthy. In addition, these results necessitate further validations by well-designed prospective clinical trials with a larger cohort.

## Figures and Tables

**Table 1:** Differences in individual variables between groups with and without fall history (*n* = 4045).

Variables		Classification	Without Fall	With Fall	χ^2^	*p*
			(*n* = 3105)	(*n* = 940)		
			*n* (%) or	*n* (%) or		
			M ± SD *	M ± SD *		
Demographic	Age		75.4 ± 6.2	76.2 ± 6.2	3.29	0.001
	Sex	Male	1047 (33.7)	233 (24.8)	26.62	<0.001
		Female	2058 (66.3)	707 (75.2)		
	Marital status	Living with spouse	1817 (58.5)	433 (46.1)	45.35	<0.001
		Living without spouse	1288 (41.5)	507 (53.9)		
	Living status	Alone	859 (27.7)	337 (35.8)	38.92	<0.001
		Living with spouse	1456 (46.9)	341 (36.3)		
		Living with children	675 (21.7)	234 (24.9)		
		Others	115 (3.7)	28 (3.0)		
Socioeconomic	Education	0–6 years	2228 (71.7)	687 (73.1)	3.99	0.263
		7–9 years	422 (13.6)	127 (13.5)		
		10–12 years	341 (11.0)	104 (11.1)		
		≥13 years	114 (3.7)	22 (2.3)		
	Quantiles of household income	Q1 (lowest)	827 (26.6)	312 (33.2)	22.84	<0.001
		Q2	745 (24.0)	182 (19.4)		
		Q3	636 (20.5)	180 (19.1)		
		Q4	517 (16.7)	134 (14.3)		
		Q5 (highest)	380 (12.2)	132 (14.0)		
Health status	Disease	Hypertension	2043 (65.8)	673 (71.6)	11.00	0.001
		Diabetes	978 (31.5)	328 (34.9)	3.81	0.051
		Dementia	78 (2.5)	33 (3.5)	2.70	0.101
		Arthritis	1495 (48.1)	517 (55.0)	13.55	<0.001
	BMI **	Underweight (<18.5)	164 (5.3)	55 (5.9)	1.66	0.646
		Normal (≥18.5, <25)	1992 (64.1)	583 (62.0)		
		Overweight (≥25)	949 (30.6)	302 (32.1)		
	Number of medication(s)	0	157 (5.0)	29 (3.1)	258.00	<0.001
		1	170 (5.5)	29 (3.1)		
		2	241 (7.8)	64 (6.8)		
		≥3	2537 (81.7)	818 (87.0)		
Health-relatedBehavior	Exercise	None	1308 (42.1)	429 (45.6)	4.63	0.099
		<150 min. a week	680 (21.9)	206 (21.9)		
		≥150 min. a week	1117 (36.0)	305 (32.5)		
	Smoking	Past/Never	2859 (92.1)	872 (92.8)	0.48	0.489
		Current	246 (7.9)	68 (7.2)		
	Drinking	None	2558 (82.4)	794 (84.5)	3.25	0.197
		≤1 standard drink/day	243 (7.8)	72 (7.6)		
		>1 standard drink/day	304 (9.8)	74 (7.9)		

* M ± SD, mean ± standard deviation. ** BMI, body mass index.

**Table 2:** Differences in physical and psychological characteristics between groups with and without fall history (*n* = 4045).

Physical and Psychological Variables		Classification	Without Fall	With Fall	χ^2^	*p*
			(*n* = 3105)	(*n* = 940)		
			*n* (%)	*n* (%)		
Physical	Visual discomfort	No	1760 (56.7)	488 (51.9)	6.64	0.010
		Yes	1345 (43.3)	452 (48.1)		
	Hearing discomfort	No	2375 (76.5)	656 (69.8)	17.26	<0.001
		Yes	730 (23.5)	284 (30.2)		
	ADL * limitation	No	2710 (87.3)	738 (78.5)	44.09	<0.001
		Yes	395 (12.7)	202 (21.5)		
	IADL ** limitation	No	1895 (61.0)	445 (47.3)	55.46	<0.001
		Yes	1210 (39.0)	495 (52.7)		
	Nutrition	Good	1222 (39.4)	284 (30.2)	25.81	<0.001
		Poor	1883 (60.6)	656 (69.8)		
Psychological	Depression	No	1944 (62.6)	473 (50.3)	45.32	<0.001
		Yes	1161 (37.4)	467 (49.7)		

* ADL, activities of daily living. ** IADL, instrumental activities of daily living.

**Table 3:** Multivariable logistic regression analysis of factors associated with fall incidents in older adults with negative subjective health status.

Variables	Model I	Model II	Model III	Model IV
	OR (95% CI)	OR (95% CI)	OR (95% CI)	OR (95% CI)
Visual discomfort	1.04 (0.89–1.22)	1.05 (0.90–1.23)	1.04 (0.89–1.21)	1.04 (0.89–1.22)
Hearing discomfort	1.31 (1.10–1.56) *	1.31 (1.10–1.56) *	1.30 (1.09–1.55) *	1.30 (1.09–1.55) *
ADL limitation	1.41 (1.14–1.74) *	1.39 (1.13–1.72) *	1.39 (1.13–1.73) *	1.40 (1.13–1.74) *
IADL limitation	1.33 (1.12–1.58) *	1.34 (1.13–1.60) *	1.34 (1.12–1.60) *	1.34 (1.13–1.61) *
Poor nutrition	1.18 (1.00–1.40) *	1.19 (1.01–1.41) *	1.12 (0.94–1.33)	1.12 (0.94–1.33)
Depression	1.39 (1.19–1.63) *	1.42 (1.21–1.66) *	1.43 (1.22–1.68) *	1.44 (1.23–1.69) *

Model I: adjusted for demographic (age, sex, marital, and living status) characteristics only. Model II: adjusted for demographic and socioeconomic (education and household income) characteristics. Model III: adjusted for demographic, socioeconomic, and health status (disease, BMI, and the number of medications) characteristics. Model IV: adjusted for demographic, socioeconomic, health status, and health-related behavior (exercise, smoking, and drinking) characteristics. * *p* < 0.05.

## Data Availability

The data presented in this study are available from the authors upon reasonable request.
